# Mechanical Properties of Auxetic Cellular Material Consisting of Re-Entrant Hexagonal Honeycombs

**DOI:** 10.3390/ma9110900

**Published:** 2016-11-07

**Authors:** Xiangwen Zhang, Deqing Yang

**Affiliations:** State Key Laboratory of Ocean Engineering, Collaborative Innovation Center for Advanced Ship and Deep-Sea Exploration, School of Naval Architecture, Ocean and Civil Engineering, Shanghai Jiao Tong University, Shanghai 200240, China; zxwsjtu@gmail.com

**Keywords:** auxetic cellular material, re-entrant honeycombs, Poisson’s ratio, relative density, scale, bearing capacity, dynamic performance

## Abstract

A preliminary study of the mechanical properties of auxetic cellular material consisting of re-entrant hexagonal honeycombs is presented. For different scales of the honeycombs, the finite element method (FEM) and experimental models are used to perform a parametric analysis on the effects of the Poisson’s ratio (cell angle) and the relative density (cell thickness) of honeycombs on bearing capacity and dynamic performance of the auxetic material. The analysis demonstrates that the ultimate bearing capacity of the presented auxetic cellular material is scale-independent when the Poisson’s ratio and the relative density are kept constant. The relationship between the geometric parameters and vibration level difference of the honeycombs is also revealed, which can be divided into two converse parts around the Poisson’s ratio v=−1.5. When v is smaller than −1.5, increasing the cell thickness leads to an increase in the vibration level difference of the honeycombs. Moreover, the dynamic performance of thin-walled honeycombs is greatly influenced by the scale of the honeycombs, especially for the ones with small Poisson’s ratio. These conclusions are verified by a frequency response test and a good agreement between the numerical results and experimental data is achieved.

## 1. Introduction

Poisson’s ratio is defined as the negative ratio between the longitudinal expansion and the lateral contraction of a material during loading [1]. Conventional materials present a positive Poisson’s ratio and their cross-sections become larger under compression and smaller under tension. Nonetheless, the classical theory of elasticity does not preclude the existence of materials with negative Poisson’s ratio, also known as “auxetic” after a 1991 study when Evans et al. re-elaborated this theory [2]. Unlike conventional materials/structures, auxetic ones present the very unusual property of becoming wider when stretched and narrower when squashed [3].

Auxetic materials constitute a new class of materials that not only can be found in nature, i.e., cubic elemental metals [4], but can also be fabricated, including honeycombs [5], polymeric and metallic foams [6], and microporous polymers [7]. These materials demonstrate unique and enhanced mechanical properties compared with their conventional counterparts [8,9]. For example, it has been shown experimentally that the indentation resistance of auxetic materials has been enhanced up to four times when compared with their conventional equivalent [10]. Other enhanced properties are mechanical hardness, toughness, and stiffness [11]. In terms of the dynamic performance, auxetic materials also show an overall superiority regarding damping and acoustic properties compared to the conventional materials [12,13]. It has been found that auxetic honeycomb structures not only show an improvement of transmission loss factors but also show an isotropic acoustic signature at lower modes and also have isotropic mechanical properties [14,15]. Owing to their lower price, easy availability, and desirable mechanical properties, auxetic materials find a broad range of applications in many industrial sectors, especially in automotive, aerospace, and marine industries [16,17]. More and more auxetic materials and structures with different microstructures are used to replace the conventional counterparts and have achieved satisfactory results [18]. For example, in the aeronautic industry, the replacement of foam cores in sandwich structures with auxetic foams results in a significant decrease in the level of noise that reaches the inside of the cabin, since the acoustic absorption properties of auxetics have been shown to be superior to those of conventional materials [19].

It has also been found that almost all of the auxetic materials are designed based on the simple mechanism that the global stiffening effects of them are determined by the unit cell [20,21]. Over the years, several different units that can achieve auxetic behavior have been proposed, such as re-entrant hexagonal honeycombs [22,23], rotating rectangles and triangles [24,25], as well as arrow-head and star-shaped configurations [26,27]. Among them, re-entrant cellular material is one of the most classical auxetic materials and has been extensively studied [28,29], since the presence of their auxetic behavior is a scale-independent property; that is, the deformation mechanism of re-entrant cellular materials can operate at any scale ranging from the nano-level to the macro-scale [30].

This paper is designed to further study the mechanical properties of auxetic cellular materials made of the foldable (the aspect ratio of honeycombs is 2), equal-height, re-entrant hexagonal honeycombs. By changing the geometric parameters of the re-entrant honeycombs, these auxetic cellular materials will take on a tunable negative Poisson’s ratio. This study focuses on the effect of the geometric parameters of the honeycombs, such as cell angle, thickness, and scale, on the bearing capacity and vibration reduction performance of the materials. The finite element method (FEM) was used as the modeling technique to obtain the static characteristics of the honeycombs and the results of the Poisson’s ratio for the honeycombs have been verified by analytical expressions. Numerical analysis and experimental verifications for the dynamic characteristics of the honeycombs were also carried out, which turned out to be in good agreement with each other.

## 2. Effects of Parameters on Bearing Capacity of the Auxetic Cellular Material

### 2.1. Finite Element Model Description

**Figure 1:**
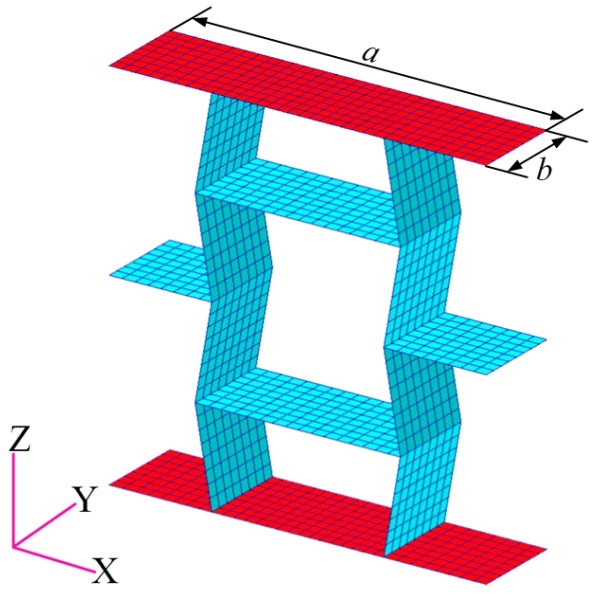
The FE model of the re-entrant hexagonal honeycomb, where the red parts are faceplates and the blue parts are honeycombs. *a* and *b* are the length and the width of the faceplate, respectively.

**Figure 2:**
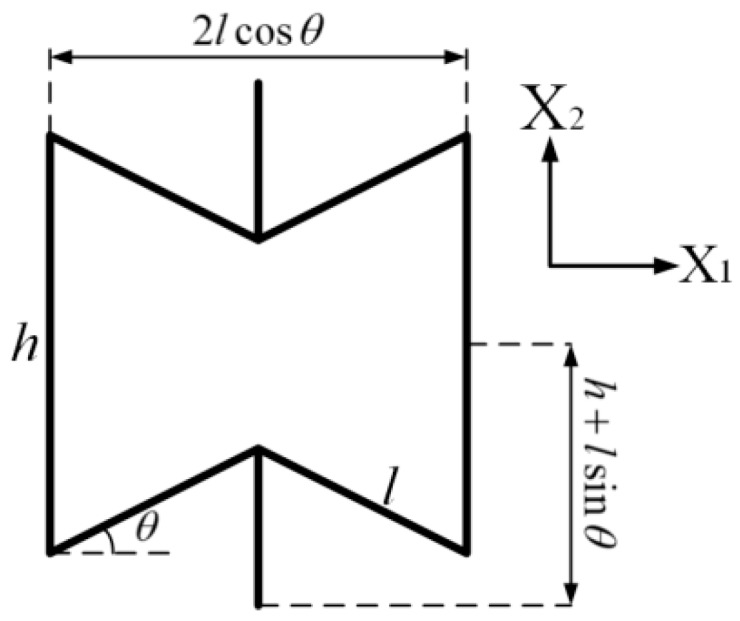
Cell geometry and the coordinate system used for the re-entrant hexagonal honeycomb, where *h* is the vertical length of the cell member, *l* is the inclined length of the cell member, and θ is the cell angle.

Finite element models of the presented samples consisting of cellular materials in this paper, as shown in Figure 1, are constructed using MSC/Patran software. The samples are made of steel faceplates and steel honeycombs with cell angles within the range −45° < θ < 0°. The geometric parameters of the honeycombs satisfy the relationship of Equation (1). For the foldable (the aspect ratio *h*/*l* of honeycombs is 2), equal-height, re-entrant hexagonal honeycombs, when the cell height 2lcosθ is kept constant, the vertical length *h* and the inclined length *l* vary with the change of the cell angle θ (refer to Figure 2). The faceplate was formed in the shape of a rectangle with the length *a* = 100 mm, the width *b* = 20 mm, and the thickness *t* = 50 mm. The loads were applied along the *z* direction and the height of the sample was 100 mm. Both the faceplates and the honeycombs were simulated as standard Quad4 elements in the finite element models with an element size of 2 mm × 2.5 mm. The material is isotropic and the properties of density, Young’s modulus, and Poisson’s ratio are $\rho_{s}=7800\;\mathrm{kg}/m^{3}$, $E_{s}=200\;\mathrm{GPa}$, and $v_{s}=0.27$, respectively. DOF1 (Degree Of Freedom), DOF2, DOF4, DOF5, and DOF6 of the transverse symmetry plane of the sample, and DOF3, DOF4, DOF5, DOF6 of the longitudinal symmetry plane of the sample, were constrained to simulate free tension and compression. The apparent in-plane mechanical properties of the honeycombs, such as Young’s modulus and Poisson’s ratio, are determined by the uniaxial loads.

(1) $$\left\{ \begin{array}{l} h=2l\\ 2l\cos\theta =const \end{array}\right.$$

### 2.2. The Accuracy of Finite Element Models

Using the geometric configuration and coordinate system of Figure 2, we put forward the hypothesis that flexing of the cell walls causes deformation of the re-entrant honeycombs when an external load is applied. Treating the cell walls as beams and using simple mechanics, the Poisson’s ratio and relative density of the cells are derived, which can be expressed as follows [25]:

(2) $$v_{21}=-\frac{\varepsilon_{1}}{\varepsilon_{2}}=\frac{\cos^{2}\theta }{(h/l+\sin\theta )\sin\theta }$$

(3) $$\frac{\rho }{\rho_{s}}=\frac{t/l(h/l+2)}{2\cos\theta (h/l+\sin\theta )}$$

According to Equations (2) and (3), as for foldable (*h*/*l* = 2), equal-height, re-entrant hexagonal honeycombs, the Poisson’s ratio of the auxetic cellular materials changes with the cell angle. Similarly, relative density can be altered by the cell thickness, inclined length, and cell angle. Poisson’s ratio of the honeycombs was calculated by the above analytical models. The calculations were based on the following parameters: $E_{s}=200\;\mathrm{GPa}$ and $v_{s}=0.27$, cell height 2lcosθ=42 mm, cell thickness *t* = 1 mm, and cell angle θ∈[−45°,0°]. Table 1 shows the comparison of Poisson’s ratio between the FE models and analytical expressions results. As can be seen from Table 1, a good agreement between the analytical expressions and the finite element models for the re-entrant honeycombs has been achieved. The maximum relative error for different cell angles is 7.3% and when the cell angle is −45°, the numerical result is almost the same as the theoretical value. The comparison proves that the hypothesis stated above is correct and the accuracy of the FE models can be guaranteed.

### 2.3. Linear Static Properties

**Figure 3:**
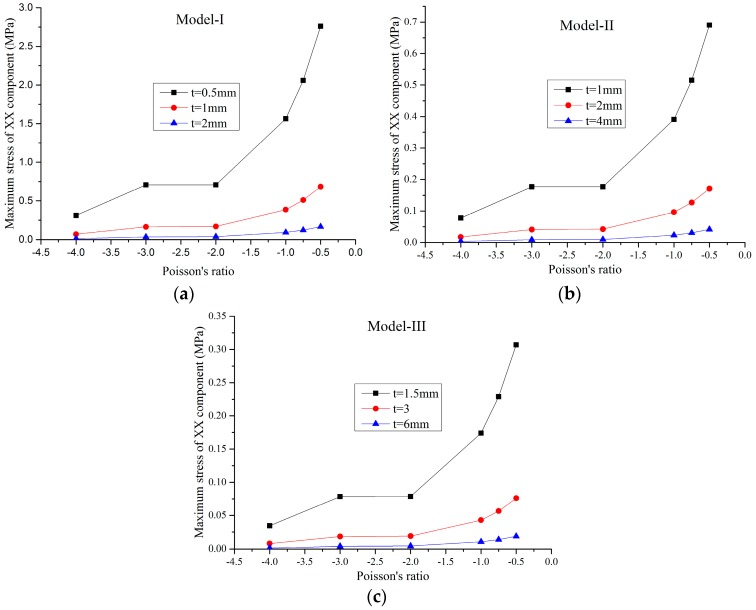
Maximum stress of the XX component versus the Poisson’s ratio: (**a**) Model-I; (**b**) Model-II; (**c**) Model-III.

**Figure 4:**
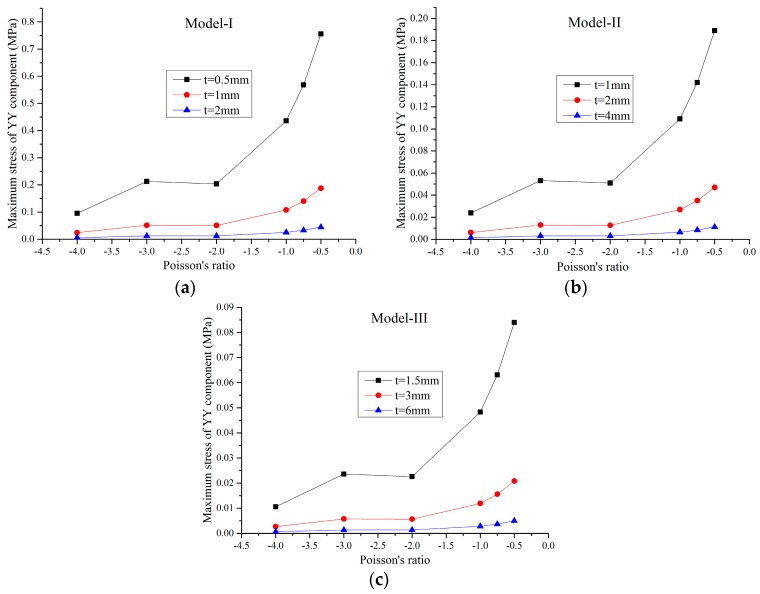
Maximum stress of the YY component versus the Poisson’s ratio: (**a**) Model-I; (**b**) Model-II; (**c**) Model-III.

**Figure 5:**
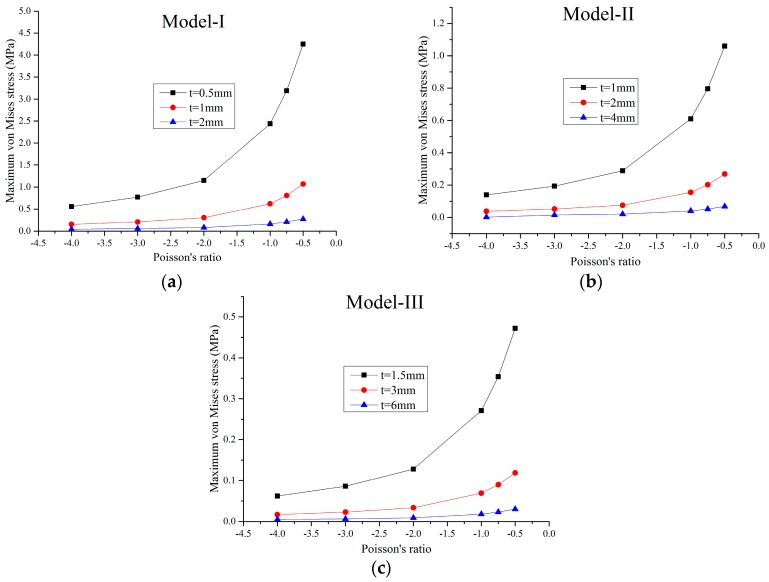
Maximum von Mises stress versus the Poisson’s ratio: (**a**) Model-I; (**b**) Model-II; (**c**) Model-III.

**Figure 6:**
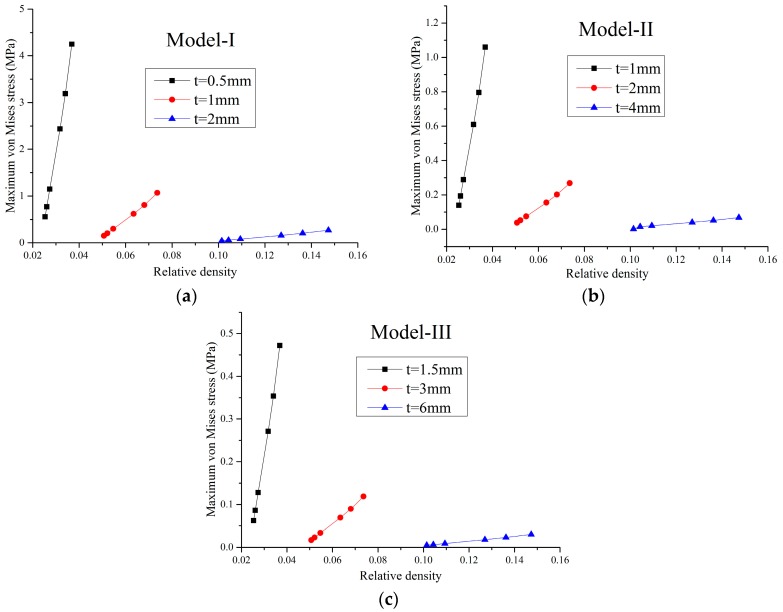
Maximum von Mises stress versus the relative density: (**a**) Model-I; (**b**) Model-II and (**c**) Model-III.

For a small load, the solid material is in the elastic range and the FE model is a linear structure. Within this elastic range, the static load of 1 N was applied on the faceplates and the maximum static von Mises stress of the FE models were obtained through the static analysis by MSC/Nastran software. In this paper, three series of FE models for different Poisson’s ratios marked as Model-I, Model-II, and Model-III, were studied. The geometric parameters of the honeycombs in these three series are proportional to each other. For example, in Model-I, there are three different FE models with the cell height 2lcosθ=42 mm and the cell thickness *t*_1_ = 0.5 mm, *t*_2_ = 1 mm and *t*_3_ = 2 mm, respectively, while in Model-II, the cell height is 84 mm and the cell thickness are 1 mm, 2 mm, and 4 mm, respectively, and so on. The effects of the Poisson’s ratio and relative density of the honeycombs on the maximum stress of the XX component, maximum stress of the YY component, and maximum von Mises stress are shown in Figure 3, Figure 4, Figure 5 and Figure 6, respectively.

In Figure 3, Figure 4 and Figure 5, it can be seen that, for a constant cell thickness, the maximum static stress of the honeycombs increases nonlinearly with the decrease of the Poisson’s ratio and the XX component stress is nearly three times more than the stress of the YY component. In Figure 6, the maximum static stress increases linearly with the increase of the relative density. The honeycombs with small thickness are much more sensitive to the Poisson’s ratio and relative density. That is, increasing the cell thickness can significantly reduce the maximum static stress of the honeycombs with small Poisson’s ratio. It also could be found that honeycombs’ zooming does not affect these conclusions. Furthermore, through the calculation of these results, an empirical formula is derived to accurately predict the maximum stress component and maximum von Mises stress of the honeycombs with different scales, which can be written as:

(4) $$\sigma_{1}=\sigma_{0}/n^{2}$$

where $\sigma_{0}$ is the maximum static stress of the initial honeycombs, $\sigma_{1}$ is the maximum static stress of the honeycombs scaling up or down, and *n* is the scaling factor.

### 2.4. Nonlinear Static Properties

There are two possible failure mechanisms for the honeycombs during compression: buckling and plastic deformation. Both of these were investigated in this paper and the results show that plastic deformation of the re-entrant honeycombs takes place before the buckling. Taking the honeycomb with cell angle θ=−45°, cell height 2lcosθ=42 mm, and cell thickness *t* = 1 mm as an example, if we do not consider the plasticity of the solid material. When the honeycomb becomes unstable, the maximum static stress is more than 300 MPa. This is impossible, because the yield point of the solid material is only 208 MPa. For other models, situations are similar to this one. Therefore, for the auxetic honeycombs in this paper, there is only one failure mechanism: plastic deformation.

**Figure 7:**
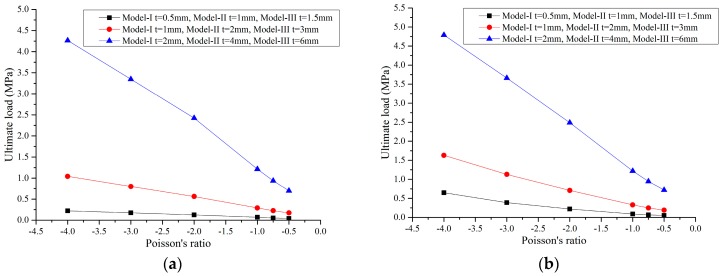
The ultimate bearing capacity of the honeycombs for different Poisson’s ratios under (**a**) compression and (**b**) tension.

In addition to the compression, when the tensile load is large enough, the plastic strain of the honeycombs will also take place. As a result, elastic-plastic properties of the honeycombs under compression and tension were studied, respectively, in this paper to forecast the ultimate bearing capacity of the cells. We assume that when the residual strain of the honeycombs is more than 0.2%, the solid material is in the plastic zone and the pressure added at this time is the ultimate load. Figure 7 shows the relationship between the Poisson’s ratio and the ultimate bearing capacity of the honeycombs.

As indicated in Figure 7, along with decreasing values of the Poisson’s ratio, the ultimate bearing capacity of the honeycombs decreases linearly under both compression and tension conditions. It can be seen that the tensile ultimate bearing capacity is higher than the compressed one and when the Poisson’s ratio is larger than −1, the cell thickness has a major influence over the ultimate bearing capacity of the honeycombs. For a constant Poisson’s ratio, the greater the cell thickness, the better the bearing capacity. A similar conclusion can be found in Figure 7 that scaling the honeycombs up or down does not affect the ultimate bearing capacity of the material. In summary, as for foldable, equal-height, re-entrant hexagonal honeycombs, when the Poisson’s ratio and relative density are kept constant, the ultimate bearing capacity of the auxetic material is a scale-independent property.

## 3. Effects of Parameters on Vibration Reduction Performance of the Auxetic Cellular Material

Vibration performance is of great practical importance, as vibration of continuous systems with constraints implies cyclic stresses and inevitable fatigue damage. Compared with traditional systems, auxetic systems decrease the propagation of vibrations more efficiently and exhibit higher dynamic stiffness [3]. Numerical analysis and experimental verifications for the vibration performance of the foldable, equal-height, re-entrant honeycombs were carried out in this section. A reasonable agreement between the finite element results and the experimental data was obtained.

### 3.1. Numerical Analysis

**Figure 8:**
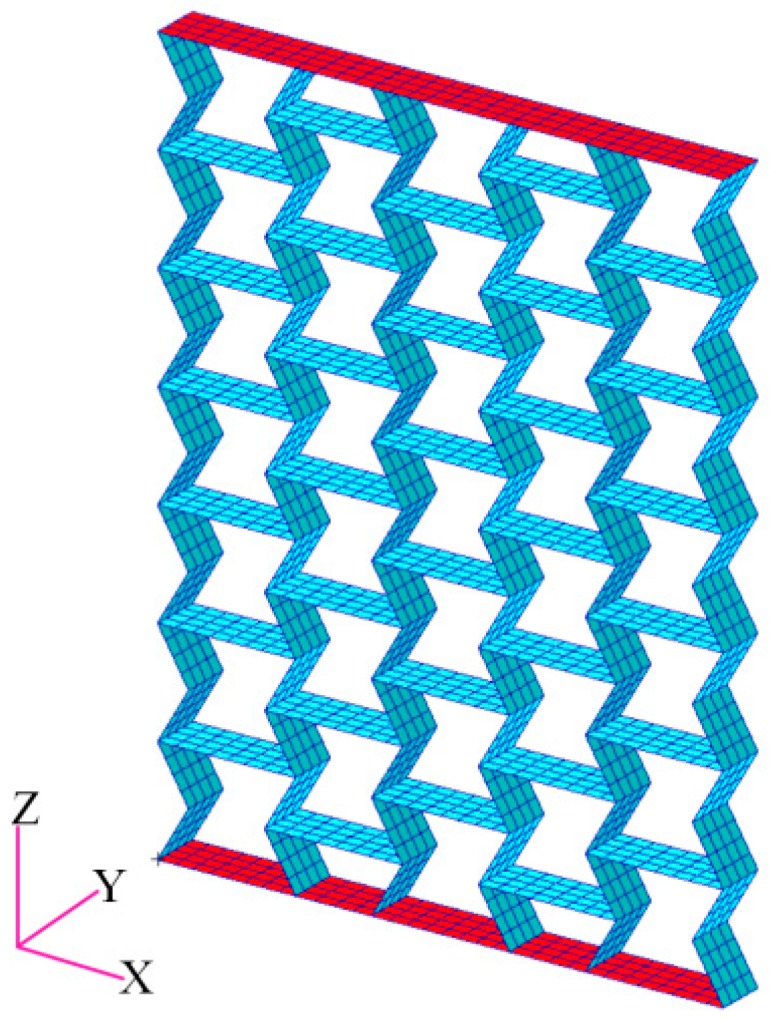
The FE model for the dynamic analysis of the auxetic honeycombs.

**Figure 9:**
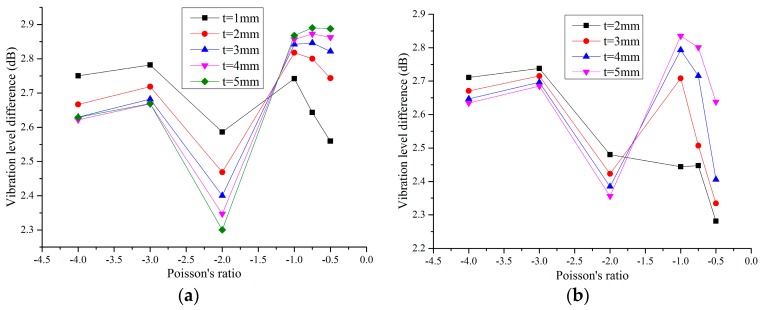
Acceleration vibration level difference of the center cell against the Poisson’s ratio for different scales: (**a**) initial models; (**b**) scale-up models.

In this section, for decreasing the boundary effect, we rebuilt the FE models to analyze the dynamic characteristics of the re-entrant hexagonal honeycombs. The models were made of seven-layer and five-row honeycombs with cell angles within the range −45° < θ < 0°, as shown in Figure 8. In order to study the influence of scales, we prepared two series of the models marked as initial models and scale-up models. The relationship between the two series is that the cell height of the latter is two times that of the former. The solid materials of the honeycombs are the same as the ones in Section 2.2. The frequency response analysis using MSC/Nastran software was done by fixing one end of the models while applying an axial unit exciting force at the other end with the sweep bandwidth of 10 Hz–100 Hz. The acceleration vibration level difference of the center cell was used to evaluate the dynamic behavior of the auxetic honeycombs, not only for the convenience of measure, but also for the decrease of error. The effects of parameters on vibration reduction performance of the re-entrant hexagonal honeycombs are shown in Figure 9.

As shown in Figure 9, for different cell thickness, the relationship between the vibration level difference and the Poisson’s ratio of the honeycombs is complex. The curves can be divided into two parts around $v_{21}=-1.5$ for both initial models and scale-up models. For the two parts, along with the decrease of the Poisson’s ratio, the vibration level difference of the center cell increases first and then decreases when the cell thickness is kept constant. When the Poisson’s ratio is larger than −1.5, the thinner the cell thickness is, the better the dynamic performance of the honeycombs will be. Conversely, when it is smaller than −1.5, increasing the cell thickness leads to an increase in the vibration level difference of the honeycombs. Moreover, changing the scale of the honeycombs has a great influence on the vibration level difference of thin-walled honeycombs, especially for the ones with a small Poisson’s ratio. For the thick-walled honeycombs, however, when the Poisson’s ratio is larger than −2, their dynamic performance is almost a scale-independent property and with the increase of the cell thickness, the differences among the models decrease.

### 3.2. Experimental Testing

**Figure 10:**
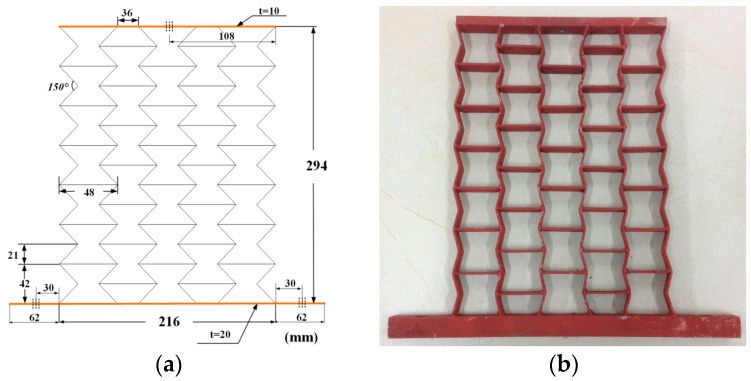
Testing samples with the cell angle θ=−15°: (**a**) geometric dimensions; (**b**) photo.

**Figure 11:**
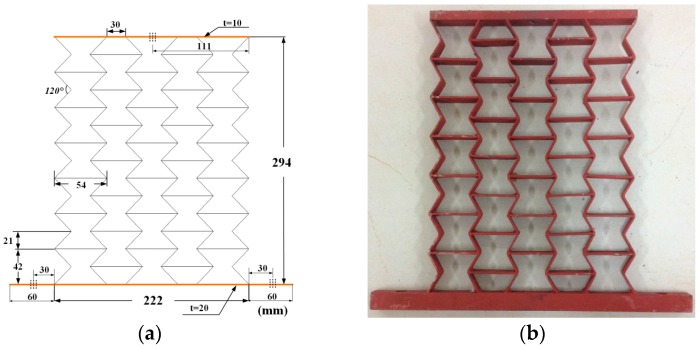
Testing samples with the cell angle θ=−30°: (**a**) geometric dimensions; (**b**) photo.

**Figure 12:**
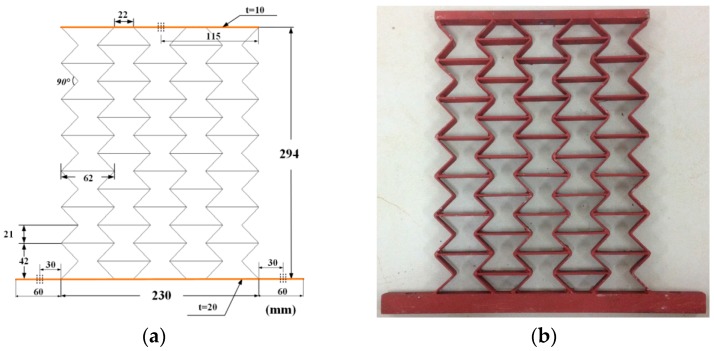
Testing samples with the cell angle θ=−45°: (**a**) geometric dimensions; (**b**) photo.

**Figure 13:**
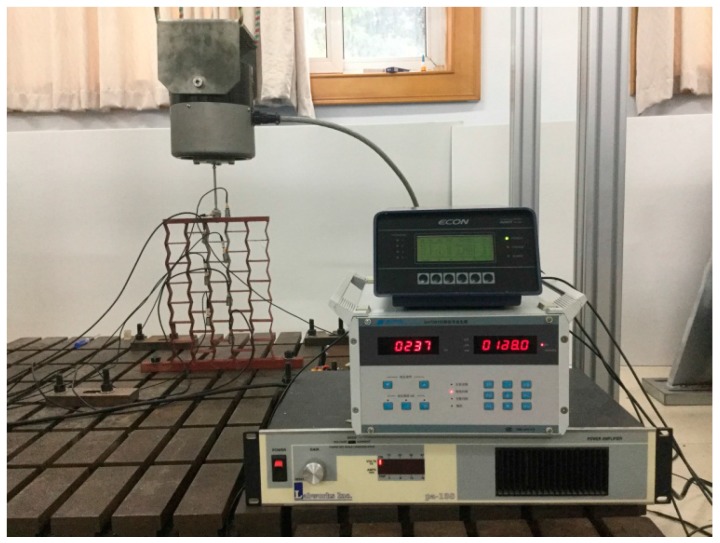
Frequency response experiment of the auxetic honeycombs.

**Figure 14:**
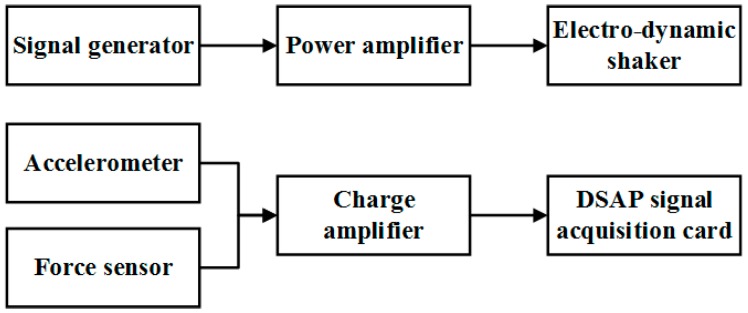
Block diagram of the excited and measured analysis system.

**Figure 15:**
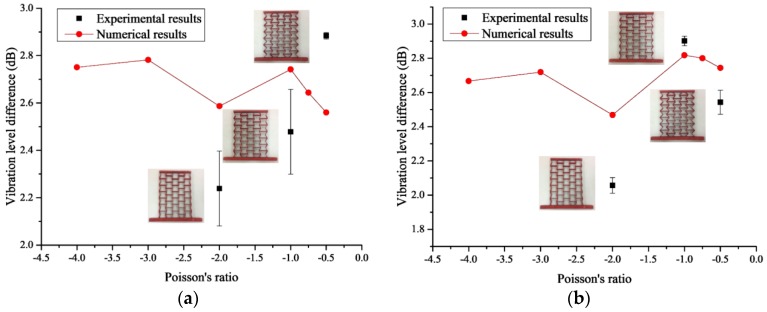
Comparison of the results between the FE models and experiments for different cell thicknesses: (**a**) *t* = 1 mm and (**b**) *t* = 2 mm.

Samples were manufactured by welding different thickness steel plates according to the blueprint, shown in Figure 10, Figure 11 and Figure 12. The parameters of the samples are consistent with the initial models mentioned in Section 2.1. For each cell angle, two samples with cell thicknesses of 1 mm and 2 mm are prepared, respectively. The samples were fixed on the test bench and loaded vertically (Figure 13). Frequency response testing was done to analyze the dynamic characteristics of the samples. The main instruments used are a signal generator (B and K 1027, B and K, Donghuatest Inc., Jingjiang, China), a power amplifier (MI-2004, ECON Technologies, Hangzhou, China), an electro-dynamic shaker (ET 139, Labworks Inc., Costa Mesa, CA, USA), a laser sensor (PSV-300F, Polytec, Beijing, China), and a DASP signal acquisition card (DH5920N, Donghuatest Inc., Jingjiang, China). In addition, accelerometers (B and K 4366, B and K, Donghuatest Inc., Jingjiang, China) and force sensors (5110, Donghuatest Inc., Jingjiang, China) were used to collect the signals of different measuring points in the frequency domain. The block diagram of the excited and measured analysis system is shown in Figure 14. Three experiments were done for each sample with different exciting forces, ranging from 4 N to 8 N with the sweep bandwidth of 10 Hz–100 Hz. The root mean square of the vibration acceleration at each frequency (10 Hz–100 Hz) was calculated with the center frequency of the 1/3 octave bands based on ISO standards. The experimental tests have been conducted at the Engineering Mechanics Experimental Center of Shanghai Jiao Tong University. The results are listed in Table 2 and Table 3 and the comparison of the results between the FE models and experiments are shown in Figure 15.

Table 2 and Table 3 show that increasing or decreasing the exciting force has a slight effect on the vibration level difference: with the increase of the exciting force, the vibration level difference increases. In fact, in the elastic range of the solid material, the model is linear and the vibration level difference will not change with the exciting forces. The main reason for this discrepancy between the theory and the experiment is that, for the limitation of the honeycomb dimensions, discontinuous welding may exist in some portions which will affect the propagation of vibrations. On the other hand, as seen in the two tables, some average values of the experiments are very close to the numerical results, and the maximum relative error is −16.69%, which exists in the model with θ=−15° and *t* = 1 mm. In Figure 13 it can be found that experimental results are around the numerical curves, which indicates that the experimental results are credible. Additionally, Figure 15 also shows that the discrete degree of the data for the models with *t* = 2 mm is much smaller. This can be attributed to the stiffness of the honeycombs. Under the same exciting forces, the greater the cell thickness is, the larger the stiffness of the honeycombs will be. The honeycombs with greater stiffness are much less easily influenced by other factors, such as constraints and loading directions during the test. One of the defects of the experiments is that the samples in this paper are made by welding, which cannot ensure the whole quality of the honeycombs. In the future, casting or integral cutting may be a better choice.

## 4. Conclusions

In this paper, the effects of the Poisson’s ratio and relative density of foldable, equal-height, re-entrant hexagonal honeycombs on the bearing capacity and vibration reduction performance of auxetic cellular materials are studied numerically and experimentally.

- For constant cell thickness, the maximum static stress of the honeycombs increases nonlinearly with the decrease of the Poisson’s ratio, and decreases linearly with the decrease of the relative density.
- Along with decreasing values of the Poisson’s ratio, the ultimate bearing capacity of the honeycombs decreases linearly under both compression and tension conditions, and the tensile ultimate bearing capacity is higher than the compressed one.
- When the Poisson’s ratio and relative density are kept constant, the ultimate bearing capacity of the auxetic materials is scale-independent.
- When the Poisson’s ratio is larger than −1.5, the thinner the cell thickness, the better the dynamic performance of the honeycombs. Conversely, when it is smaller than −1.5, increasing the cell thickness leads to an increase in the vibration level difference of the honeycombs. Moreover, changing the scale has a great influence on the dynamic performance of thin-walled honeycombs, especially for the ones with small Poisson’s ratio.
- To improve the accuracy of experiments, casting or integral cutting technologies are suggested for the manufacture of experimental samples. In the near future, work will focus on the acoustic characteristics and dispersive properties of the materials.

## Figures and Tables

**Table 1:** Comparison of the Poisson’s ratio between the FE models and analytical expressions.

**Cell Angle**	θ=−7.5°	θ=−10°	θ=−15°	θ=−30°	θ=−37°	θ=−45°
**FE Model**	−3.71	−2.88	−2.06	−1.06	−0.80	−0.49
**Theory**	−4.00	−3.00	−2.00	−1.00	−0.75	−0.50
**Relative Error**	−7.3%	−4.0%	3.2%	6.1%	7.0%	−0.2%

**Table 2:** Experimental results of the vibration level difference under different exciting forces with the cell thickness *t* = 1 mm (dB).

Force	4 N	6 N	8 N	Average	Relative Error with FEM Result
θ=−15°	1.78	2.18	2.75	2.24	−13.44%
θ=−30°	2.04	2.34	3.05	2.48	−9.61%
θ=−45°	2.73	2.91	3.01	2.88	12.64%

**Table 3:** Experimental results of the vibration level difference under different exciting forces with the cell thickness *t* = 2 mm (dB).

Force	4 N	6 N	8 N	Average	Relative Error with FEM Result
θ=−15°	1.83	1.99	2.34	2.06	−16.69%
θ=−30°	2.73	2.85	3.13	2.90	2.98%
θ=−45°	2.27	2.46	2.90	2.54	−7.32%
